# Chemical Pressure‐Induced Unconventional Band Convergence Leads to High Thermoelectric Performance in SnTe

**DOI:** 10.1002/advs.202409735

**Published:** 2024-11-07

**Authors:** Hongwei Ming, Zhong‐Zhen Luo, Zhigang Zou

**Affiliations:** ^1^ Fujian Science & Technology Innovation Laboratory for Optoelectronic Information of China Fuzhou Fujian 350108 P. R. China; ^2^ Key Laboratory of Advanced Materials Technologies International (HongKong Macao and Taiwan) Joint Laboratory on Advanced Materials Technologies College of Materials Science and Engineering Fuzhou University Fuzhou 350108 P. R. China; ^3^ State Key Laboratory of Photocatalysis on Energy and Environment Fuzhou University Fuzhou 350116 P. R. China; ^4^ Eco‐materials and Renewable Energy Research Center College of Engineering and Applied Sciences Nanjing University Nanjing 210093 P. R. China; ^5^ National Laboratory of Solid State Microstructures Nanjing University Nanjing 210093 P. R. China

**Keywords:** band convergence, chemical pressure, fermi velocity, phonon scattering, SnTe

## Abstract

Band convergence is considered a net benefit to thermoelectric performance as it decouples the density of states effective mass ($m_{d}^{*}$) and carrier mobility (*µ*) by increasing valley degeneracy. Unlike conventional methods that typically prioritize $m_{d}^{*}$ at the expense of *µ*, this study theoretically demonstrates an unconventional band convergence strategy to enhance both $m_{d}^{*}$ and *µ* in SnTe under pressure. Density functional theory calculations reveal that increasing pressure from 0 to 5 GPa moves the Σ‐band of SnTe upward, reducing the energy offset between L‐ and Σ‐band from 0.35 to 0.2 eV while preserving the light band feature of the L‐band. Consequently, a high power factor (*PF*) of 119.2 µW cm^−1^ K^−2^ at 300 K is achieved for p‐type SnTe under 5 GPa. Chemical pressure also induces conduction band convergence, significantly enhancing the *PF* of n‐type SnTe. Additionally, the interplay between pressure‐induced phonon modes leads to a moderate increase in lattice thermal conductivity of SnTe below 3 GPa, which combined with the significantly enhanced *PF*, contributes to a large enhancement in *ZT*. Consequently, predicted *ZT* values of 2.12 at 650 K and 2.55 at 850 K are obtained for p‐ and n‐type SnTe, respectively, showcasing substantial performance enhancements.

## Graphical abstract

Unlike conventional methods that typically prioritize valley degeneracy at the expense of carrier mobility, this study theoretically demonstrates an unconventional band convergence strategy to enhance both valley degeneracy and carrier mobility in SnTe under pressure. Furthermore, ultrahigh *ZT* values of 2.12 at 650 K and 2.55 at 850 K are theoretically predicted for p‐ and n‐type SnTe under 5 GPa.

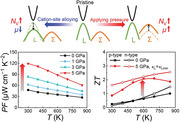

## 1 Introduction

Thermoelectric materials, which directly convert waste heat to useful electricity without hazardous emissions or moving parts, have garnered much attention.^[^ ^1^ ^]^ The energy conversion efficiency of thermoelectric materials is governed by the dimensionless figure of merit *ZT* = *S* ^2^ *σT*/(*κ*_L_ + *κ*_e_), where *S*, *σ*, *T*, *κ*_L_, and *κ*_e_ denote Seebeck coefficient, electrical conductivity, absolute temperature, lattice thermal conductivity, and charge carrier thermal conductivity, respectively. The *ZT* of an optimally doped material is directly proportional to the thermoelectric quality factor, *B*, which is determined by weighted mobility divided by the lattice thermal conductivity (*µ*_w_/*κ*_L_).^[^ ^2^ ^]^ Over the years, two main strategies have been employed to enhance thermoelectric performance. One approach focuses on increasing *µ*_w_ by achieving band convergence,^[^ ^3^ ^]^ forming resonant states,^[^ ^4^ ^]^ or realizing multiband synglisis.^[^ ^5^ ^]^ The other aims to reduce the *κ*_L_ through the introduction of all‐scale hierarchical architectures,^[^ ^6^ ^]^ off‐center doping,^[^ ^7^ ^]^ lattice softening,^[^ ^8^ ^]^ or exploring compounds with strong lattice anharmonicity.^[^ ^9^ ^]^

Lead‐free SnTe is an intriguing alternative to PbTe for mid‐temperature thermoelectric applications due to its analogous rock‐salt crystal structure and electronic band structure.^[^ ^4^, ^10^ ^]^ However, the large energy offset (Δ*E*_V_ = 0.35 eV) between the light (L‐band) and heavy valence band (Σ‐band)^[^ ^3^, ^6^ ^]^ has restricted enhancement in *µ*_w_ and consequently thermoelectric performance. To achieve band convergence, researchers have utilized experimental trial‐and‐error methods or density functional theory (DFT)‐based calculations to identify potential dopants. For example, researchers have used temperature‐dependent Hall coefficient (*R*_H_) measurements to identify the band convergence.^[^ ^10^, ^11^ ^]^ Tan et al.^[^ ^11a,b^ ^]^ found that 3% MnTe alloying reduces the peak temperature of *R*_H_ (convergence temperature, *T*_peak_) of SnTe from 770 to 620 K,^[^ ^11a^ ^]^ while 2% HgTe alloying reduces the *T*_peak_ of Sn_0.98_Bi_0.02_Te to 573 K.^[^ ^11b^ ^]^ Yang et al.^[^ ^11c^ ^]^ demonstrated that 2% V doping decreases the *T*_peak_ of SnTe to 560 K. On the other hand, Tan et al.^[^ ^12^ ^]^ combined DFT calculation with an s‐p bonding model to investigate the effects of cation‐site substituting in SnTe for achieving band convergence. They highlighted the crucial role of the s orbital of dopants in facilitating band convergence and identified Be, Zn, Cd, Hg, Mg, and Mn as promising candidates.^[^ ^12^ ^]^

Despite great efforts that have been devoted to achieving improved band convergence in SnTe, the Δ*E*_V_ or the convergence temperature is yet high, severely limiting thermoelectric performance enhancement across a wide temperature range. Even worse, previous methods to achieve conventional band convergence (BC)^[^ ^10^, ^12^, ^13^ ^]^ by reducing the dispersion of the L‐band have frequently sacrificed carrier mobility (*µ*). Since *µ*_w_ is related to *µ* through *µ*_w_ = *µ* ${\left(m_{d}^{*}/m_{e}\right)}^{3/2}$= *µN*_V_ ${\left(m_{b}^{*}/m_{e}\right)}^{3/2}$, where *N*_V_ denotes valley degeneracy, $m_{d}^{*}$ represents density of states effective mass, and $m_{b}^{*}$ denotes single band effective mass. Moreover, for isotropic bands and charge carriers predominately scattered by acoustic phonons, *µ* is inversely proportionally to 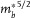. Therefore, achieving an enhanced *N*_V_ by compromising the small $m_{b}^{*}$ of the L‐band definitely reduces *µ*. Thus, regulating the Σ‐band to achieve unconventional band convergence (UBC) while preserving the high *µ* feature of the L‐band is a novel strategy, which, even though challengeable, is vital to achieving high *µ*_w_ and *ZT*.

Using DFT calculations, this study showcases a UBC in SnTe achieved by applying chemical pressure. Specifically, as pressure increases, the Σ‐band composed of Sn‐s and Te‐p antibonding states shifts upward while maintaining the morphology of the L‐band, leading to a reduction in Δ*E*_V_. Subsequently, Δ*E*_V_ decreases from 0.35 to 0.2 eV as pressure increases from 0 to 5 GPa. Moreover, calculated electrical transport properties indicate that this UBC results in a simultaneous improvement in $m_{d}^{*}$ and *µ*, thereby significantly enhancing *S*, *σ*, and the corresponding power factor (*PF* = *S* ^2^ *σ*). At 300 K and under 5 GPa pressure, a large *PF* of 119.2 µW cm^−1^ K^−2^ was achieved for p‐type SnTe, which is 1.1 times higher than that under ambient conditions. Similarly, applying pressure induces conduction band convergence by decreasing the energy of degenerate deep bands and significantly enhances the *PF* of n‐type SnTe, reaching a *PF* of 126.9 µW cm^−1^ K^−2^ under 5 GPa. Additionally, this study theoretically analyzed pressure‐dependent phonon transport characteristics in SnTe. The interplay between pressure‐induced softening of transverse acoustic phonon modes and hardening of longitudinal acoustic and optical modes leads to a moderate increase in *κ*_L_ below 3 GPa. As a result, ultrahigh *ZT* values of 2.12 at 650 K and 2.55 at 850 K are predicted for p‐ and n‐type SnTe under 5 GPa, respectively.

## 2 Results and Discussion

### 2.1 Structural Properties

**Figure 1:**
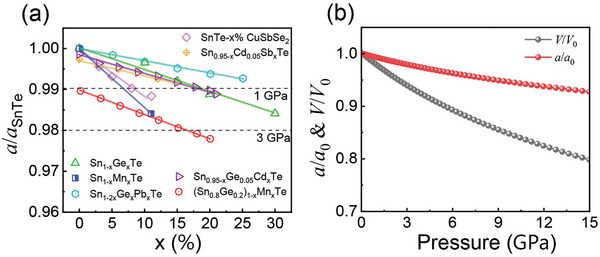
a) Variation of the normalized lattice parameter (*a*/*a*_SnTe_) of SnTe‐based materials^[^ ^11^, ^15^ ^]^ with doping/alloying content (x). b) Pressure‐dependent normalized lattice parameter (*a*/*a*_0_) and volume (*V*/*V*_0_) of SnTe.

Heavy doping/alloying is a common strategy to improve the thermoelectric performance of SnTe by regulating band structure or introducing additional phonon scattering centers.^[^ ^11^, ^15^ ^]^ However, the concurrent effects of lattice shrinkage (as shown in **Figure** **1**a), referred to as chemical pressure, on thermoelectric transport properties have been overlooked. The shrinkage of the lattice constant under pressure obviously shortens the average bond lengths, directly influencing the electronic band structure and related transport properties. This motivated us to theoretically investigate the pressure‐dependent carrier‐phonon transport properties in the SnTe system.

By fitting the total ground state energy with respect to the volume using the Birch–Murnaghan equation of state (Figure S1, Supporting Information), the variation of normalized lattice parameter (*a*) and volume (*V*) with pressure can be determined, as shown in Figure 1b. According to Figure 1b, ≈1% and 2% lattice contraction can induce high chemical pressure up to 1 and 3 GPa in SnTe, respectively. Moreover, a previous study reported that SnTe undergoes pressure‐driven phase transitions at 5 GPa.^[^ ^14^ ^]^ Therefore, to avoid this phase transition, theoretical calculations of SnTe were conducted under hydrostatic pressures of 0, 1, 3, and 5 GPa within the *Fm* $\bar{3}$ *m* space group.

### 2.2 Electronic Structures

**Figure 2:**
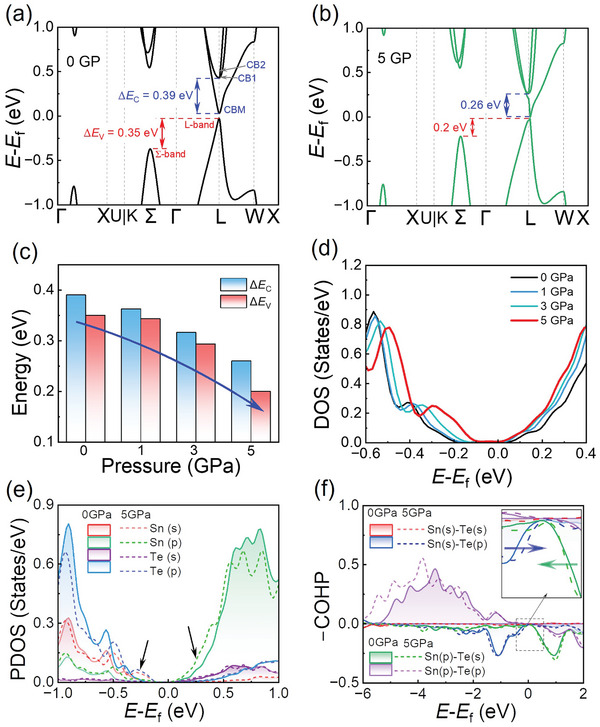
Band structures of SnTe under a) 0 GPa and b) 5 GPa. c) Variations of Δ*E*_C_ and Δ*E*_V_ with pressure. d) The density of states (DOS) of SnTe under 0, 1, 3, and 5 GPa. e) Projected density of states (PDOS) and f) the crystal orbital Hamiltonian populations (COHP) of SnTe under 0 and 5 GPa.

**Figure** **2**a is the band structure of SnTe under 0 GPa, showing a direct band‐gap semiconductor characteristic. The valence band maximum (VBM) is located at the L point of the Brillouin zone (denoted as L‐band). In contrast, a sub‐valence band is situated at the Σ point (denoted as Σ‐band), with an energy difference (Δ*E*_V_) between L‐ and Σ‐band ups to 0.35 eV. The conduction band minimum (CBM) is also located at the L point, exhibiting an energy difference (Δ*E*_C_) of 0.39 eV between the two degenerate deep conduction bands (CB1 and CB2). In Figure 2b, as pressure increases, the Σ‐band moves upward, and CB1 and CB2 move downward, resulting in the convergence of the valence and conduction bands, respectively. Figure 2c shows that Δ*E*_V_ and Δ*E*_C_ decrease with increasing pressure. Specifically, as pressure increases from 0 to 5 GPa, Δ*E*_V_ decreases from 0.35 to 0.2 eV, and Δ*E*_C_ decreases from 0.39 to 0.26 eV. These band convergences enhance the density of states (DOS) slope around VBM and CBM (Figure 2d), thereby boosting the $m_{d}^{*}$ and *S* of both p‐ and n‐type SnTe.

**Figure 3:**
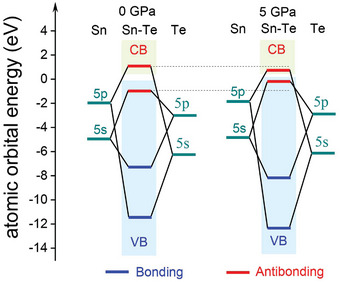
The schematic molecular orbital diagram illustrates Sn‐Te bonding in SnTe under conditions of 0 and 5 GPa, respectively.

To uncover the underlying mechanism of this pressure‐driven band convergence in SnTe, we compared the partial density of states (PDOS) and crystal orbital Hamiltonian populations (COHP) of SnTe under 0 and 5 GPa. Positive values of ‐COHP suggest bonding interactions that stabilize the structure, while negative ‐COHP values signify antibonding interactions destabilizing it. Figure 2e shows that the VBM is composed of Sn (s) and Te (p) orbitals, whereas the CBM is primarily contributed by the Sn (p) and Te (s) orbitals. Figure 2f confirms through COHP analysis that both VBM and CBM are governed by antibonding states. A schematic molecular orbital diagram of Sn‐Te bonding is provided in **Figure** **3**. Furthermore, Figure 2f reveals that increasing pressure shifts these antibonding states to the band edge. Specifically, as pressure increases, the Sn (s)‐Te (p) antibonding states move upward and contribute to the Σ‐band (Figure S2, Supporting Information), causing the Σ‐band to shift upward faster than that of the L‐band, thereby achieving band convergence.

**Figure 4:**
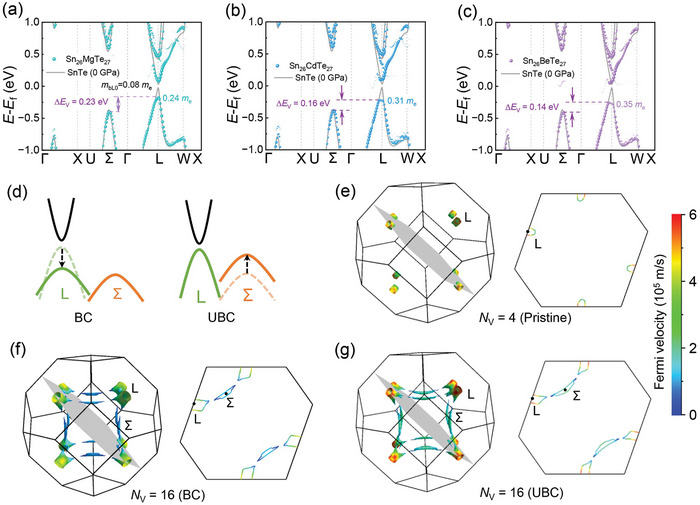
Comparisons of the effective band structures of a) Sn_26_MgTe_27_, b) Sn_26_CdTe_27_, and c) Sn_26_BeTe_27_ with SnTe under 0 GPa. d) A schematic diagram of conventional band convergence (BC) and unconventional band convergence (UBC) in SnTe. 3D carrier pocket visualization and the corresponding sectional view using band energy isosurfaces of 0.25 eV below the valence band maximum (VBM). Different colors represent the magnitude of Fermi velocity: e) SnTe under 0 GPa, f) SnTe with 5% lattice expansion, and g) SnTe under 5 GPa.

It should be noted that the effects of pressure‐induced UBC in SnTe differ significantly from BC achieved in p‐type SnTe through cation‐site alloying (**Figure** **4**a–c) or lattice expansion (Figure S3, Supporting Information). Here, Mg, Cd, and Be are predicted by Tan et al.^[^ ^12^ ^]^ to be effective dopants for achieving valence BC in SnTe. This BC is achieved by reducing the dispersion of the L‐band (band flattening) to lower the energy of the top of the L‐band. Specifically, Mg, Cd, and Be alloying decreases the Δ*E*_V_ of SnTe from 0.35 to 0.23, 0.16, and 0.14 eV, respectively. However, this also increases the effective mass of the L‐valence ($m_{\mathrm{bL}}^{*}$) from 0.08 to 0.24, 0.31, and 0.35 *m*_e_, respectively.

For materials with multi‐bands/valleys that participate in transport and charge carriers predominately scattered by acoustic phonons, the *µ* and $m_{d}^{*}$ of the host can be described by the following equations:^[^ ^16^ ^]^

(1) $$\mu =\sum_{i}\frac{p_{i}e\tau_{i}}{p_{\mathrm{tot}}m_{\mathrm{bi}}^{*}}=\sum_{i}\frac{p_{i}}{p_{\mathrm{tot}}}\mu_{i}$$

(2) $$\mu_{i}\;\propto \;{\left(m_{\mathrm{bi}}^{*}\right)}^{-5/2}$$

(3)

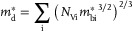

here, $m_{\mathrm{bi}}^{*}$, *µ*_i_, *p*_i_, and *τ*_i_ are the single band effective mass, carrier mobility, carrier concentration, and relaxation time of the i‐th energy band/valley. The *p*_tot_ is the total carrier concentration in the host. *N*_Vi_ denotes valley degeneracy of the i‐th energy band/valley. Thus, BC involves activating the flattened L‐band (with a large $m_{\mathrm{bL}}^{*}$) for transport, which definitely reduces *µ* of the SnTe (Figure S4, Supporting Information) while improving $m_{d}^{*}$. In contrast, UBC achieved by regulating the Σ‐band can maintain the morphology and light band feature of the L‐band (Figures 4d and 2) and improve effective valley degeneracy, leading to a synergistic optimization of *µ* and $m_{d}^{*}$. The impacts of these band convergence strategies on *µ* were analyzed using the two‐band Kane model (Figure S4, Supporting Information).^[^ ^17^ ^]^

Figure 4e–g show 3D carrier pockets and the corresponding sectional views using band energy isosurfaces of 0.25 eV below the VBM. Figure 4e shows that under 0 GPa, SnTe contains eight half‐pockets located around the L points of the Brillouin zone, indicating an *N*_V_ of 4. Additionally, different colors represent the magnitude of Fermi velocity (*V*_F_). The sectional view in Figure 4e clearly shows that the *V*_F_ values reach 6 × 10^5^ m s^−1^ around the L point. Figure 4f exhibits eight half‐pockets around the L points and twelve full‐pockets around the Σ point in the Brillouin zone, resulting in an *N*_V_ of 16 for SnTe with 5% lattice expansion. The sectional view in the right part of Figure 4f also highlights these additional hole pockets. Conversely, the *V*_F_ values of the carriers in the L half‐pockets show a significant drop (≈4.3 × 10^5^ m s^−1^), originating from the L‐band flattening (Figure S3b, Supporting Information). Figure 4g displays hole pockets of SnTe under 5 GPa, illustrating that applying pressure increases the *N*_V_ of SnTe to 16 while maintaining high *V*_F_ features of the L‐band. Electron pocket visualizations of SnTe under 0 and 5 GPa were provided in Figure S5 (Supporting Information), indicating that applying pressure can elevate the conduction band degeneracy (*N*_C_) from 4 to 12 in SnTe. Meanwhile, the high *V*_F_ feature of the L‐conduction band remains unaffected by pressure. Moreover, comparing the morphologies of electron pockets (Figure S5e,f, Supporting Information) and hole pockets (Figure 4e) of SnTe under 0 GPa, electron pockets appear more complex than hole pockets, suggesting potentially higher n‐type thermoelectric properties compared to p‐type, with optimized carrier concentration.

Further DFT calculations indicate that applying pressure (lattice shrinkage) can regulate the sub‐valence band of other IV–VI compounds, such as PbTe (space group: *Fm* $\bar{3}$ *m*), PbSe (space group: *Fm* $\bar{3}$ *m*), PbS (space group: *Fm* $\bar{3}$ *m*), GeTe (space group: *Fm* $\bar{3}$ *m*), GeSe (space group: *Fm* $\bar{3}$ *m*), and SnSe (space group: *Pnma*). Notably, pressure induces valence and conduction band convergence of GeTe (space group: *Fm* $\bar{3}$ *m*) and SnSe (space group: *Pnma*), as shown in Figures S6 and S7 (Supporting Information), suggesting that this strategy can also be used to regulate the band structure of other thermoelectric materials.

### 2.3 Electrical Transport Properties

The calculation of the electrical transport properties within Boltzmann theory requires a carrier relaxation time (*τ*), which is a function of both temperature and carrier concentration. In order to fully evaluate the electrical transport properties of SnTe, we calculate the *τ* using the deformation potential method. The calculated *σ* (*T*) curves fit well with the experimental results, as shown in Figure S8 (Supporting Information), indicating the validity of the deformation potential method for predicting *σ* in SnTe.

Figures S9 and S10 (Supporting Information) present the dependencies of *σ*, *S*, and *PF* on carrier concentration (*p* for holes and *n* for electrons) and temperature. At 300 K, the *σ* of p‐type SnTe (*p* = 2 × 10^20^ cm^−3^) increases from 4.7 × 10^5^ to 10.7 × 10^5^ S m^−1^ as pressure increases from 0 to 5 GPa. Similarly, n‐type SnTe (*n* = 2 × 10^20^ cm^−3^) shows an increase from 2.4 × 10^5^ to 3.8 × 10^5^ S m^−1^ under the same conditions. Temperature‐dependent *σ* under pressures of 1, 3, and 5 GPa (Figure S10a,b, Supporting Information) indicate significant enhancements across all investigated temperatures compared to that of SnTe under ambient pressure. This improvement at fixed carrier concentrations is attributed to increased *µ*, originating from enhanced valley degeneracy and maintained L band edge morphology.

Figure S9c,d (Supporting Information) are the carrier concentration‐dependent *S* of p‐type and n‐type SnTe, demonstrating that increasing pressure enhances the *S* at a given *p*/*n*. For instance, at 300 K and *p* = 1 × 10^20^ cm^−3^, the *S* of p‐type SnTe increases from 73 to 134 µV K^−1^ with increasing pressure from 0 to 5 GPa. Correspondingly, the absolute value of *S* for n‐type SnTe rises from 112 to 149 µV K^−1^ under the same conditions (Figure S9d, Supporting Information). Temperature‐dependent trends in Figure S10c,d (Supporting Information) further highlight that the absolute value of *S* increases with increasing pressure. These enhancements in *S* should be attributed to UBC elevated $m_{d}^{*}$.

**Figure 5:**
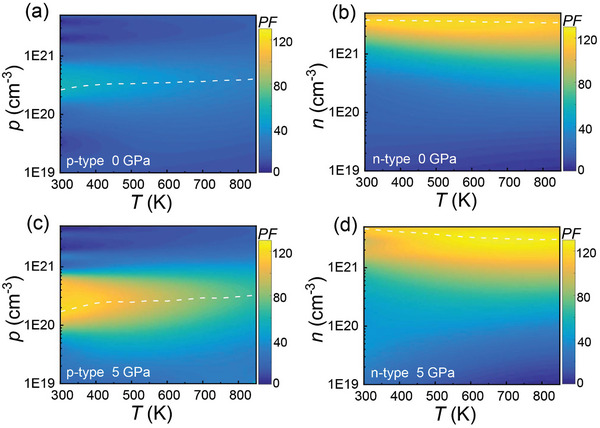
Variations of the power factor (*PF*) with carrier concentration and temperature of SnTe; the different colors represent the magnitude of *PF* in units of µW cm^−1^ K^−2^. a) p‐type and b) n‐type of SnTe under 0 GPa; c) p‐type and d) n‐type of SnTe under 5 GPa. The white dashed lines represent the optimal carrier concentration (*p*_opt_ for holes and *n*_opt_ for electrons) necessary to achieve the *PF*_max_.

The synergistic improvement in *σ* and *S* leads to a substantial enhancement in the *PF* for both p‐type and n‐type SnTe, as shown in **Figure** **5** and Figure S11 (Supporting Information). The different colors represent the magnitude of *PF* values. The white dashed lines represent the optimal carrier concentration (*p*_opt_ for holes and *n*_opt_ for electrons) required to maximize *PF* at various temperatures. Specifically, under 0 GPa, the *p*_opt_ of SnTe is between 2.6 × 10^20^ and 4 × 10^20^ cm^−3^, while under 5 GPa, it reduces to 1.7 × 10^20^–3.2 × 10^20^ cm^−3^, as shown in Figure S12a (Supporting Information). This *p*_opt_ is directly influenced by the Δ*E*_V_ and $m_{d}^{*}$. Specifically, under 5 GPa, SnTe with a smaller Δ*E*_V_ needs a lower *p*_opt_ to activate the Σ‐bands participating in transport compared to SnTe under 0 GPa. Additionally, the pressure‐induced UBC significantly enhances the *PF*_max_ of p‐type SnTe. For instance, at 300 K, the *PF*_max_ of SnTe under 0 GPa is 57.8 µW cm^−1^ K^−2^ (*p* = 2.87 × 10^20^ cm^−3^), whereas under 5 GPa, it increases to 119.2 µW cm^−1^ K^−2^ (*p* = 1.9 × 10^20^ cm^−3^), as shown in Figure 5a,c.

In contrast, the *n*_opt_ for n‐type SnTe under different pressures is ultrahigh, reaching up to ≈3.5 × 10^21^ cm^−3^ (Figure S12b, Supporting Information), owing to the large Δ*E*_C_ and high $m_{d}^{*}$ of its conduction bands. The *PF*_max_ of n‐type SnTe under 0 GPa is 119.6 µW cm^−1^ K^−2^ (*T* = 500 K, *p* = 3.49 × 10^21^ cm^−3^), increasing to 126.9 µW cm^−1^ K^−2^ (*T* = 700 K, *p* = 3.0 × 10^21^ cm^−3^) under 5 GPa, as shown in Figure 5b,d, showing a moderate enhancement. Due to intrinsic acceptor‐like defects such as Sn vacancies in SnTe,^[^ ^18^ ^]^ achieving *n* as high as 11 × 10^21^ cm^−3^ is challenging experimentally. Therefore, enhancing the *PF* of n‐type SnTe at *n* below 1 × 10^21^ cm^−3^ is of particular importance. Figure 5d, Figures S9f and S10f (Supporting Information) highlight that increasing pressure can significantly improve the *PF* of n‐type with *n* below 1 × 10^21^ cm^−3^.

Furthermore, the *PF*_max_ of n‐type SnTe (119.6 µW cm^−1^ K^−2^) under 0 GPa surpasses that of the p‐type counterpart (57.8 µW cm^−1^ K^−2^) due to the more complex electron pockets compared to the hole pockets. Despite the low thermoelectric performance of current n‐type SnTe,^[^ ^19^ ^]^ it is anticipated that optimizing the carrier concentration will enable the realization of superior n‐type thermoelectric performance in SnTe.

### 2.4 Phonon Transport Properties

**Figure 6:**
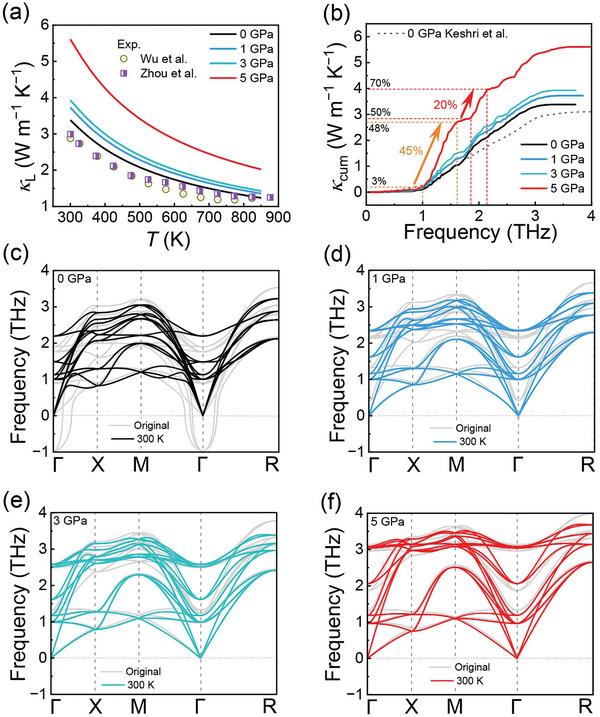
a) Temperature‐dependent lattice thermal conductivity, *κ*_L_, with experimental data indicated by dots for comparison.^[^ ^6^, ^20^ ^]^ b) Frequency‐dependent the cumulative lattice thermal conductivity (*κ*_cum_) at 300 K. Calculated phonon dispersions at 0 K (original) and 300 K of SnTe under pressures: c) 0 GPa, d) 1 GPa, e) 3 GPa, and f) 5 GPa.

To evaluate the pressure‐dependent *ZT* of SnTe, the temperature‐ and pressure‐dependent *κ*_L_ were calculated in this section. **Figure** **6**a shows that the calculated *κ*_L_ of SnTe under 0 GPa is consistent well with the experimental results reported by Wu et al.^[^ ^6b^ ^]^ and Zhou et al.^[^ ^20^ ^]^ The slight deviation (∼11% at 300 K) between the experimental and the calculated values should be attributed to additional phonon scattering from the intrinsic Sn vacancies and grain boundaries in the polycrystalline SnTe. Furthermore, Figure 6a shows a moderate enhancement in *κ*_L_ of SnTe with increasing pressure from 0 to 3 GPa. When the pressure reaches 5 GPa, the *κ*_L_ of SnTe at 300 K reaches 5.6 W m^−1^ K^−1^, which is approximately 65% higher than that of SnTe under 0 GPa.

The cumulative thermal conductivity (*κ*_cum_) as a function of phonon frequency provides valuable insights into the contribution of phonons of different frequencies to total *κ*_L_. Figure 6b shows the *κ*_cum_ versus phonon frequency at 300 K for SnTe under different pressures (0, 1, 3, and 5 GPa). The solid black curve closely matches previously reported results (dashed black curve), showing an increasing trend with phonon frequency up to ∼3 THz. Notably, phonons below 1 THz contribute only ∼3% to the total *κ*_L_ of SnTe across pressures from 0 to 5 GPa, suggesting that heat transport in SnTe is not dominated by phonons within this range. Moreover, compared to SnTe under 0 GPa, *κ*_cum_ under 5 GPa exhibits significant enhancement in the 1 to 1.6 THz and 1.85 to 2.15 THz frequency ranges. Specifically, phonons in these ranges contribute 45% and 20% to the total *κ*_L_ of SnTe under 5 GPa. Thus, the pressure‐dependent characteristics of phonon transport in these frequency bands largely contribute to the enhanced *κ*_L_ of SnTe under 5 GPa.

Figure 6c–f show the phonon dispersions of SnTe under 0, 1, 3, and 5 GPa. Figure 6c reveals distinct imaginary phonon modes at the Γ point in the original phonon dispersion, originating from the unstable cubic rock‐salt phase induced by Sn^2+^ distortion at 0 K.^[^ ^21^ ^]^ This phase remains stable at 300 K, as indicated by the absence of imaginary phonon modes in the dispersions. Figure S13 (Supporting Information) compares the phonon density of states (PhDOS) of Sn and Te in SnTe under different pressures, showing that Sn atoms dominantly contribute to PhDOS below 2 THz, while Te contributes mainly above 2 THz. Furthermore, the contribution of Sn atoms to low‐frequency phonons gradually diminishes with increasing pressure.

**Figure 7:**
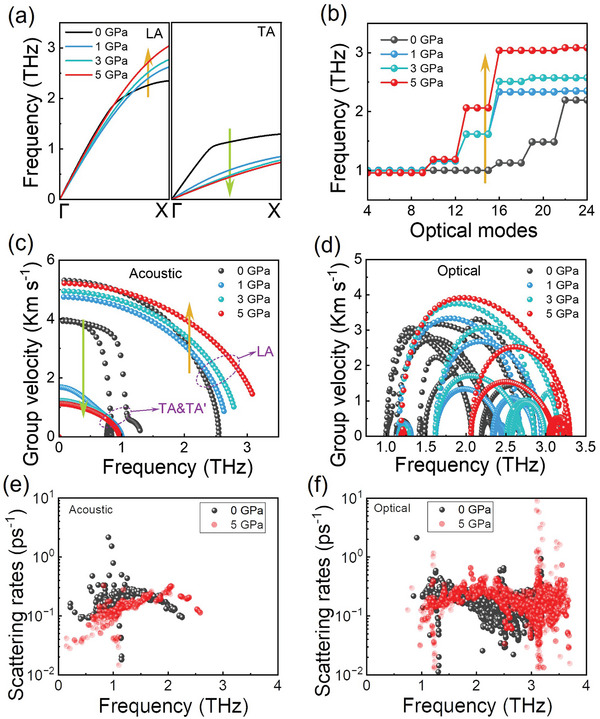
Comparisons of a) longitudinal acoustic (LA) and transverse acoustic (TA) modes of SnTe along the Γ‐X direction and b) optical models at zone center for SnTe under 0, 1, 3, and 5 GPa. Group velocities of c) acoustic modes and d) optical modes along Γ‐X direction. Comparisons of e) acoustic and optical f) modes scattering rates for SnTe under 0 and 5 GPa.

**Figure** **7**a compares the longitudinal acoustic (LA) and transverse acoustic (TA) modes of SnTe under various pressures. The LA modes above 2 THz undergo hardening with increasing pressure, leading to higher acoustic cut‐off frequencies and group velocities (Figure 7c), thereby enhancing *κ*_L_. Conversely, the low‐frequency (<2 THz) LA and TA modes soften with pressure, reducing group velocities (Figure 7c) and acoustic phonon scattering (Figure 7e; Figure S14, Supporting Information), while increasing phonon lifetimes (Figure S15, Supporting Information).

Figure 7b displays the optical modes at the zone center (Γ point) for SnTe at 0, 1, 3, and 5 GPa. Increasing pressure induces hardening in the optical modes, raising their frequencies at the Γ point, which enhances group velocities (Figure 7d) and decreases acoustic‐optical phonon scattering rates (Figure 7f; Figure S14, Supporting Information). Overall, the interplay between pressure‐induced TA mode softening and LA/optical mode hardening moderately increases *κ*_L_ below 3 GPa. Upon reaching 5 GPa, weakened acoustic‐optical phonon scattering in the frequency range of ≈1–2 THz predominantly contributes to the ≈65% enhancement in *κ*_L_.

Although the increase in *κ*_L_ is not beneficial for achieving high *ZT*, it's noteworthy that this drawback can be offset by a significant enhancement in the *PF*, particularly for SnTe under pressures below 3 GPa. Additionally, as mentioned earlier, pressure can be applied either physically or through heavy doping/alloying. The latter method can significantly enhance phonon scattering due to mass or strain field fluctuations caused by dopants, thereby optimizing *κ*_L_ concurrently. Figure S16 (Supporting Information) compares reported experimental *κ*_L_ values of SnTe‐based materials, indicating that *κ*_L_ can be minimized (*κ*_Lmin_ = 0.4 W m^−1^ K^−1^) through doping/alloying. Therefore, while the prediction of maximum *ZT* using *κ*_Lmin_ is not entirely rigorous, it is still meaningful to consider the contribution of substituent‐induced phonon scattering under an ideal condition.

### 2.5 Figure of Merit

**Figure 8:**
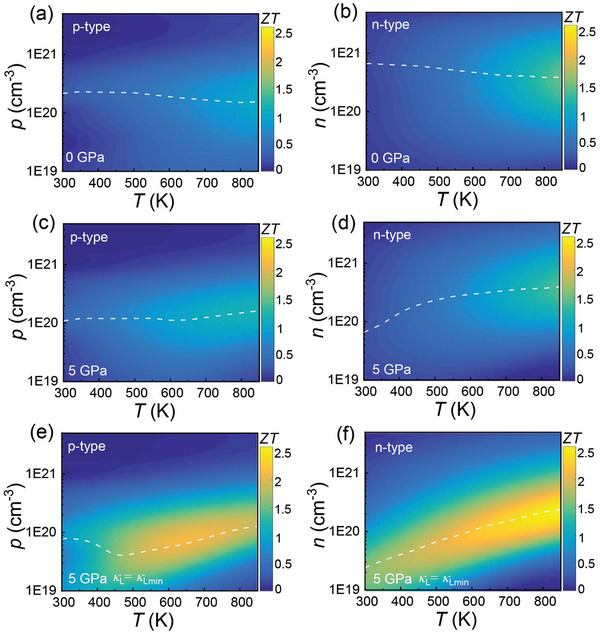
Predicted *ZT* as a function of function of temperature and carrier concentration. a) p‐type and b) n‐type SnTe under 0 GPa. c) p‐type and d) n‐type SnTe under 5 GPa. e) p‐type and f) n‐type SnTe under 5 GPa using *κ*_L_ = *κ*_Lmin_ (0.4 W cm^−1^ K^−2^). The different colors represent the magnitude of *ZT*. The white dashed lines represent the optimum carrier concentration (*p*_opt_ for holes and *n*_opt_ for electrons) for achieving the *ZT*_max_.

**Figure 9:**
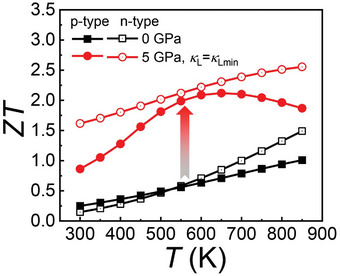
Predicted *ZT* of SnTe under 0 GPa and SnTe under 5 GPa with *κ*_L_ = *κ*_Lmin_ (0.4 W cm^−1^ K^−2^).

**Figure** **8** and Figure S17 (Supporting Information) show the *ZT* of p‐type and n‐type SnTe as a function of temperature and carrier concentration. The different colors represent the magnitude of *ZT*, with the lighter colors indicating the higher values. Figure 8 reveals that applying 5 GPa pressure effectively enhances the *ZT* of SnTe, particularly near room temperature, promoting higher averaged *ZT* (*ZT*_ave_). Furthermore, with increasing pressure, the optimum carrier concentrations *p*_opt_ and *n*_opt_ (white dashed lines) for p‐ and n‐type SnTe to achieve *ZT*_max_ decrease. This reduction benefits from UBC, enhancing *PF* at low *n*/*p*. Particularly for n‐type SnTe, *n*_opt_ decreases from approximately 3.8–6.7 × 10^20^ cm⁻^3^ to ≈0.6–3.9 × 10^20^ cm⁻^3^, facilitating high *ZT* achievement at lower carrier concentrations. Using *κ*_L_ = *κ*_Lmin_, the predicted peak *ZT* for p‐type and n‐type SnTe reaches 2.12 at 650 K and 2.55 at 850 K (Figure 8e,f), respectively. Moreover, both p‐type and n‐type SnTe under 5 GPa exhibit *ZT* values >1 over a wide range of temperatures and carrier concentrations, indicating the potential for high *ZT*_ave_. **Figure** **9** presents *ZT* values of SnTe under 5 GPa with optimal *p*_opt_ and *n*_opt_, revealing ultrahigh *ZT*_ave_ (300–850 K) of 1.8 and 2.1 for p‐type and n‐type SnTe, respectively.

## 3 Conclusion

In this work, we theoretically demonstrate an UBC approach to enhance both $m_{d}^{*}$ and *µ* of SnTe under pressure, in contrast to the conventional method that typically prioritizes $m_{d}^{*}$ at the expense of *µ*. DFT calculations reveal that increasing pressure from 0 to 5 GPa causes the heavy valence band (Σ‐band) governed by Sn (5s)‐Te (5p) antibonding states to shift upward. Simultaneously, the energy offset between the L‐ and the Σ‐band decreases from 0.35 to 0.2 eV, maintaining the light band characteristics of the L‐band. Consequently, a high *PF* of 119.2 µW cm⁻¹ K⁻^2^ was achieved at 300 K for p‐type SnTe under 5 GPa, which is ≈1.1 times higher than that of SnTe under 0 GPa. Similarly, pressure also induces conduction band convergence by decreasing the energy of the deep conduction bands, leading to a large enhancement in the *PF* of n‐type SnTe. Furthermore, the interplay between the softening of transverse acoustic phonon modes and the hardening of longitudinal acoustic/optical modes under pressure moderately increases *κ*_L_ of SnTe under pressure below 3 GPa. As a result, large *ZT* values of 2.12 at 650 K and 2.55 at 850 K are predicted for p‐type and n‐type SnTe, respectively.

## Conflict of Interest

The authors declare no conflict of interest.

## Supporting information



Supporting Information

## Data Availability

The data that support the findings of this study are available from the corresponding author upon reasonable request.
