# Supplementary material for: Chemical Pressure‐Induced Unconventional Band Convergence Leads to High Thermoelectric Performance in SnTe
Source: Adv Sci (Weinh). 2024 Nov 7;12(1):2409735. doi: 10.1002/advs.202409735 (PMC11714321; doi:10.1002/advs.202409735)
Supplement: Supplementary file 1 — Supporting Information [file ADVS-12-2409735-s001.pdf]

ADVANCED SCIENCE
Open Access

Supporting Information

for *Adv. Sci.*, DOI 10.1002/advs.202409735

Chemical Pressure-Induced Unconventional Band Convergence Leads to High Thermoelectric Performance in SnTe

Hongwei Ming, Zhong-Zhen Luo and Zhigang Zou*

Supporting Information

Chemical pressure-induced unconventional band convergence leads to high thermoelectric performance in SnTe

Hongwei Ming,[1] Zhong-Zhen Luo,[1,2,3,*] Zhigang Zou[1,2,4,5]

[1]Fujian Science & Technology Innovation Laboratory for Optoelectronic Information of China, Fuzhou, Fujian 350108, China

[2]Key Laboratory of Advanced Materials Technologies, International (HongKong Macao and Taiwan) Joint Laboratory on Advanced Materials Technologies, College of Materials Science and Engineering, Fuzhou University, Fuzhou, 350108, P. R. China.

[3]State Key Laboratory of Photocatalysis on Energy and Environment, Fuzhou University, Fuzhou 350116, China

[4]Eco-materials and Renewable Energy Research Center, College of Engineering and Applied Sciences, Nanjing University, Nanjing, 210093, China

[5]National Laboratory of Solid State Microstructures, Nanjing University, Nanjing 210093, China.

Corresponding authors: zzluo@fzu.edu.cn (Z. Z. Luo)

1. Computational details

1.1 Electronic structures and electrical transport properties

Electronic structure calculations were performed using the Vienna ab initio Simulation Package (VASP) with the projector augmented wave (PAW) method.[1] The generalized gradient approximation (GGA) with Perdew-Burke-Ernzerhof (PBE) was chosen for the exchange-correlation energy.[2] To simulate the electronic structures of SnTe under different pressures (0, 1, 3, and 5 GPa), the lattice parameters were artificially adjusted according to the pressure-dependent lattice parameter (Figure 1b). The atomic positions of SnTe under different pressures (0, 1, 3, and 5 GPa), the $Sn_{26}MgTe_{27}$, $Sn_{26}CdTe_{27}$, and $Sn_{26}BeTe_{27}$ were relaxed until the calculated Hellmann-Feynman forces on each atom were less than 0.02 eV/Å. To avoid the effect of volume shrinkage on the band structure of doped-SnTe, the volume of the doped supercell models was fixed. Structure optimization and electronic structure calculations employed an energy convergence criterion of 1×10^{-5} eV, with a plane-wave cutoff energy of 500 eV.

The band unfolding technique (VASPKIT[3] code) was utilized to clarify the effective band structure along the high symmetry directions of the primitive cell.[4] The crystal orbital Hamiltonian populations (COHP) were extracted from the plane wave using LOBSTER.[5] Visualization of carrier pockets in SnTe under different pressures was achieved using the FermiSurfer code.[6]

The temperature- and carrier concentration-dependent electrical transport properties (σ/τ, and S) were calculated using the BoltzTraP code.[7] The calculation of τ in SnTe utilized the deformation potential method,[8] where the deformation potential (E_d) was defined as $E_d = \Delta E/(\Delta V/V)$, with ΔE representing the energy change of the band extrema and $\Delta V/V$ the volume dilation. The transport effective mass (m_I^*) was determined as $m_I^* = \sigma/ne^2\tau$ and obtained directly from the BoltzTraP code.[7] The single band effective mass m_b^* is determined by analyzing the band features around the valence band maximum along three directions ($\vec{\alpha}$ = x, y, or z). It is calculated using the formula: $m_b^* = \left(m_x^* m_y^* m_z^*\right)^{1/3}$, $m_\alpha^* = \hbar^2/(\partial^2 E(k)/\partial^2 k_\alpha)$ $(\alpha = x, y, z)$.

1.2 Lattice dynamic properties

The lattice dynamical properties of SnTe under pressures of 0, 1, 3, and 5 GPa were calculated using the finite displacement method implemented in the Phonopy code.[9] Supercells of Sn_4Te_4 with a 2 × 2 × 2 structure and varying lattice parameters were constructed to realize the convergence of Hellmann-Feynman forces. The GGA-PBE was selected to determine exchange-correlation energy. Strict convergence criteria of 10^{-8} eV for energy, 10^{-7} eV Å^{-1} for atomic forces, and a plane-wave cutoff energy of 500 eV were applied to obtain accurate phonon frequencies. Temperature-dependent phonon dispersions and density of states for Sn_4Te_4 under different pressures were obtained through the ab initio molecular dynamics (AIMD) simulations within the isothermal–isovolumic canonical ensemble at 300 K. Renormalized phonon dispersions were derived using DynaPhoPy.[10]

To determine the κ_L, second-order force constants were obtained using the finite displacement method in the Phonopy code.[9] Third-order force constants, describing anharmonic phonons, were computed using the thirdorder.py code.[11] The ShengBTE package[12] was then utilized to solve the all-phonon Boltzmann transport equation and derive the κ_L.

2. Structural properties

The equilibrium energy (E_0 = –3.78 eV), the unit-cell volume (V_0 = 263.5 Å^3) at zero pressure, the bulk module (B_0 = 40.97 GPa) and its pressure derivative (B_0' = 4.18) were determined by fitting the calculated total energy as a function of unit cell volume (Figure S1) to the Birch-Murnaghan equation of state given by the following formulas:[13]

$$E(V) = E_0 + \frac{9V_0B_0}{16}\left\{\left[\left(\frac{V_0}{V}\right)^{2/3} - 1\right]^3 B_0' + \left[\left(\frac{V_0}{V}\right)^{2/3} - 1\right]^2 \left[6 - 4\left(\frac{V_0}{V}\right)^{2/3}\right]\right\} \quad \text{s1)}$$

Where V is the unit cell volume. The pressure dependent-volume (Figure 1b) was obtained by substituting the fitted B_0, B_0', and V_0 in the following formula:[13-14]

$$P(V) = \frac{3B_0}{2}\left[\left(\frac{V_0}{V}\right)^{7/3} - \left(\frac{V_0}{V}\right)^{5/3}\right]\left\{1 + \frac{3}{4}\left(B_0' - 4\right)\left[\left(\frac{V_0}{V}\right)^{2/3} - 1\right]\right\} \quad \text{s2)}$$

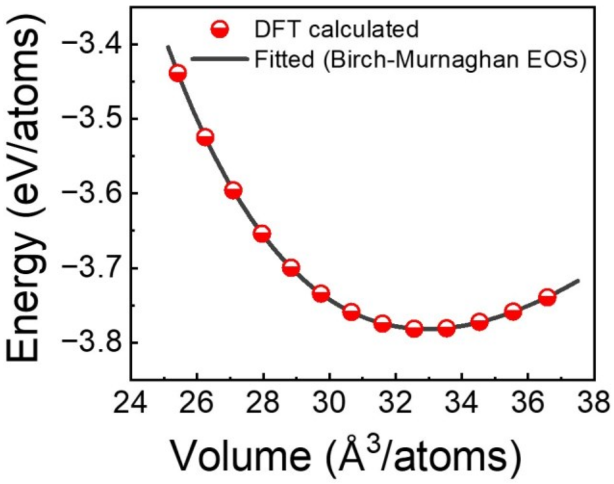

Figure S1. The variation of ground state energy of SnTe with volume. The dots are the calculated results. The solid line is the fitted result using the third-order Birch-Murnaghan equation of state (EOS).

3. Electronic structures

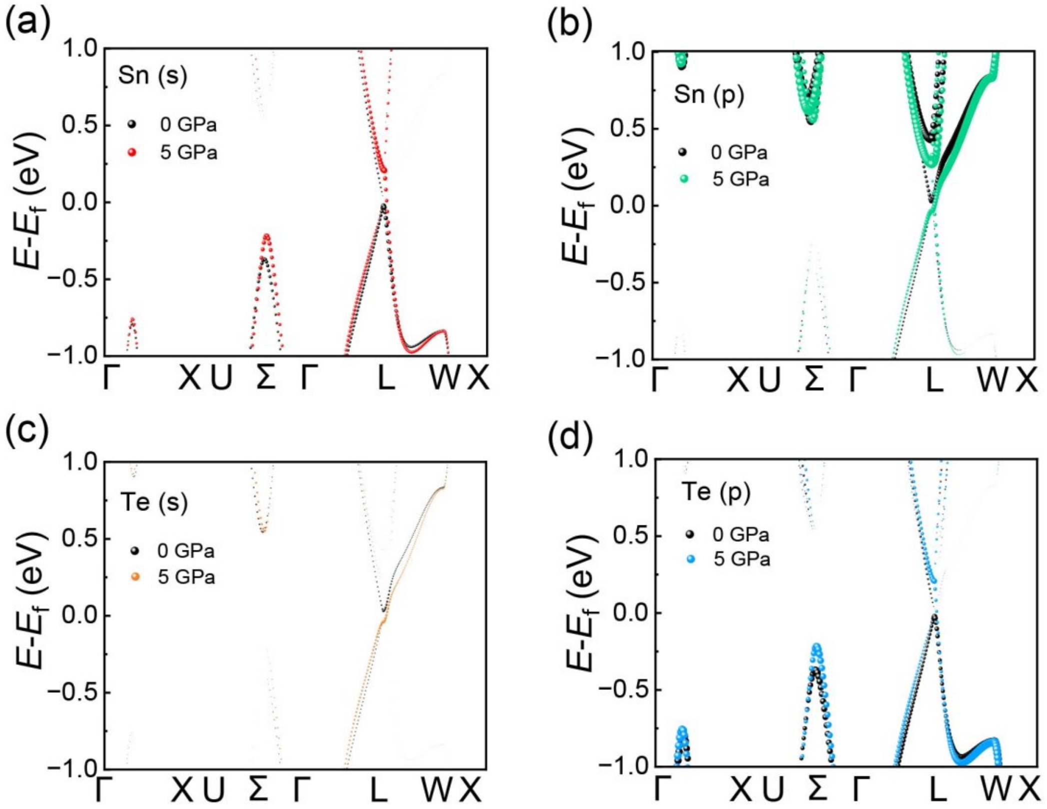

Figure S2. Orbital projected band structure of SnTe under 0 and 5 GPa. (a) Sn (s) orbitals, (b) Sn (p) orbitals, (c) Te (s) orbitals, and (d) Te (p) orbitals.

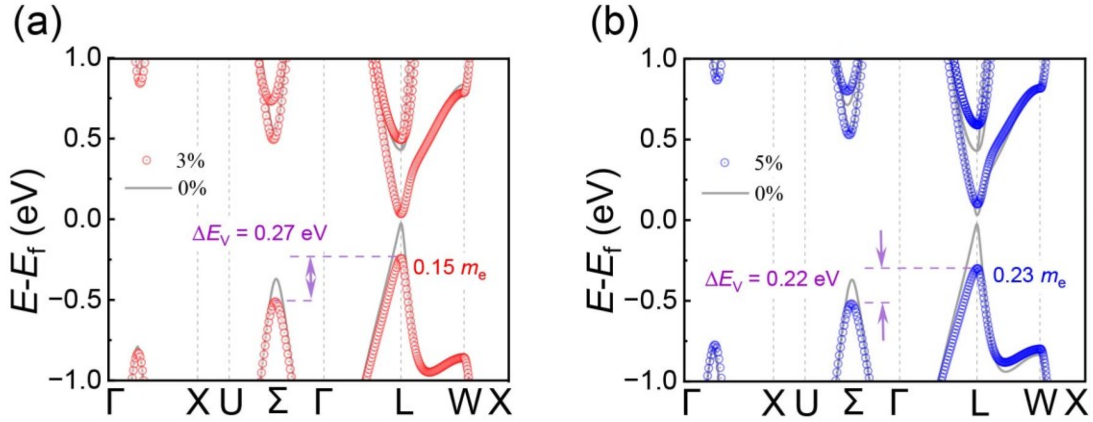

Figure S3. Band structures of SnTe with (a) 3% and (b) 5% lattice expansion.

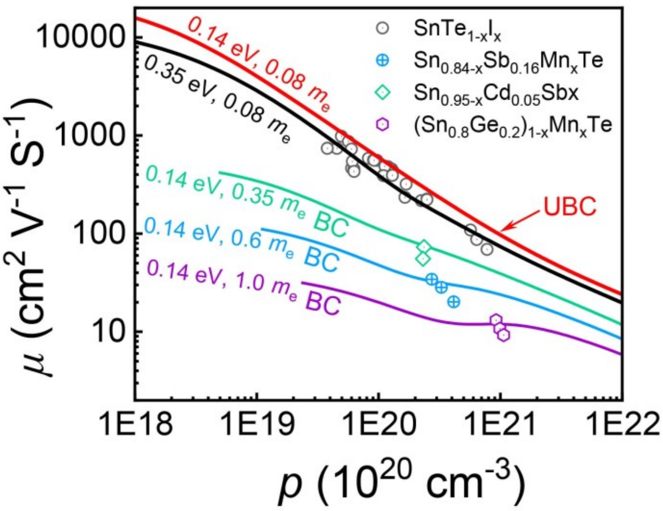

Figure S4. Carrier concentration dependent-carrier mobility. The dots represent experimental results[15], while the curves are the calculated results using the two-band Kane model[15a, 16] with different ΔE_V and m^*_{bL}. The black curve, with ΔE_V = 0.35 eV and m^*_{bL} = 0.08 m_e, shows the calculated μ for pristine SnTe, aligning well with experimental data from I-doped SnTe samples.[15a] The red curve, obtained with ΔE_V = 0.14 eV and m^*_{bL} = 0.08 m_e (unconventional band convergence, UBC), indicates an enhancement in μ. In contrast, the green, blue, and purple curves, calculated with decreased ΔE_V and increased m^*_{bL} (conventional band convergence, BC) compared to pristine SnTe, demonstrate a large reduction in μ.

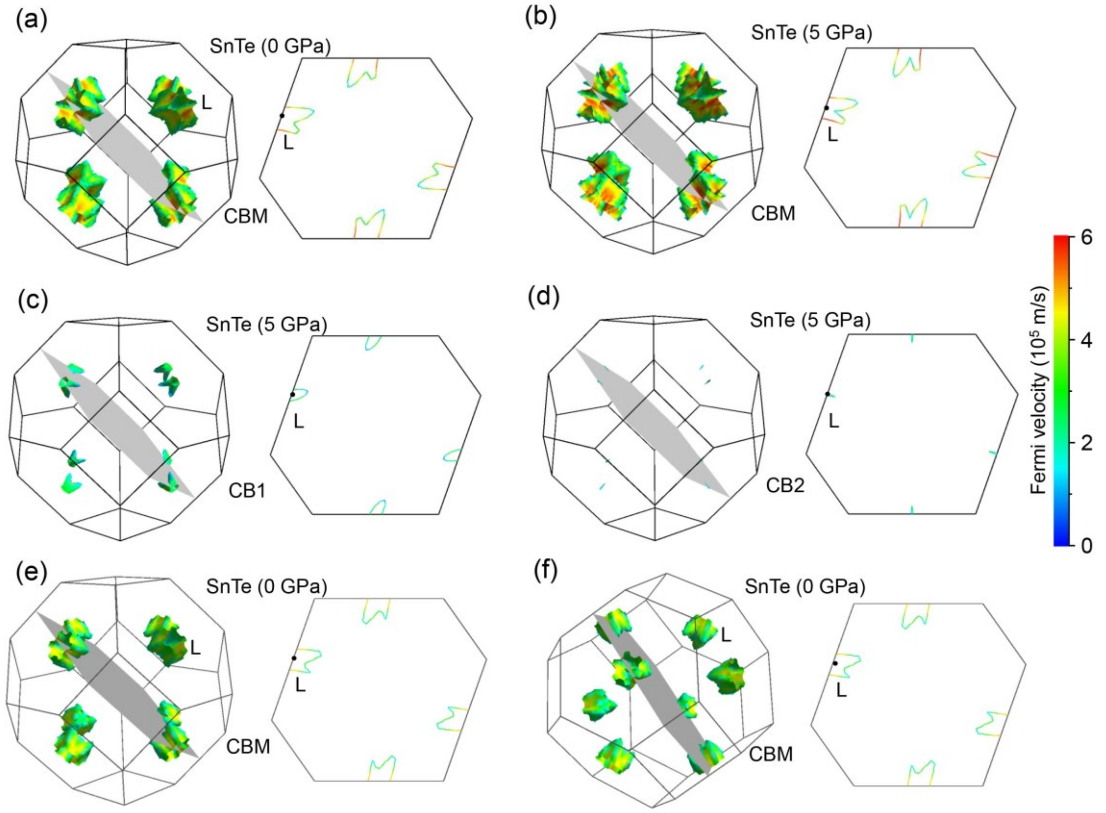

Figure S5. Three-dimensional carrier pocket visualization and the corresponding sectional view using band energy isosurfaces 0.3 eV above the conduction band minimum (CBM). (a) SnTe under 0 GPa, (b) the CBM, (c) CB1, and CB2 of SnTe under 5 GPa. Three-dimensional carrier pocket visualization and the corresponding sectional view using band energy isosurfaces 0.25 eV above the CBM. (e) and (f) show electron pockets of SnTe under 0 GPa from different angles. The different colors represent the magnitude of Fermi velocity.

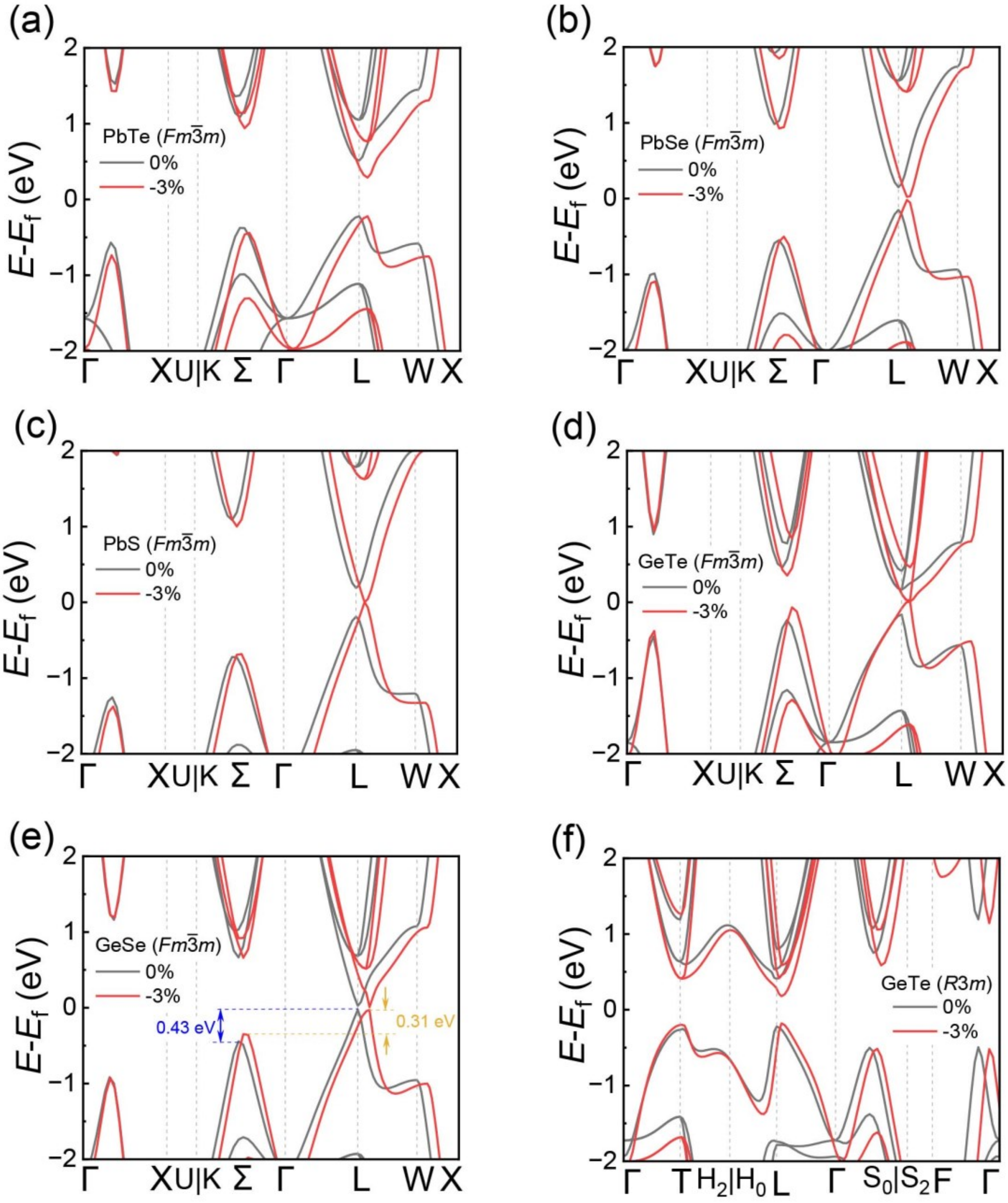

Figure S6. Comparisons of band structures with 0% and -3% strain. (a) PbTe (space group: $Fm\bar{3}m$), (b) PbSe (space group: $Fm\bar{3}m$), (c) PbS (space group: $Fm\bar{3}m$), (d) GeTe (space group: $Fm\bar{3}m$), (e) GeSe (space group: $Fm\bar{3}m$), and (f) GeTe (space group: $R3m$).

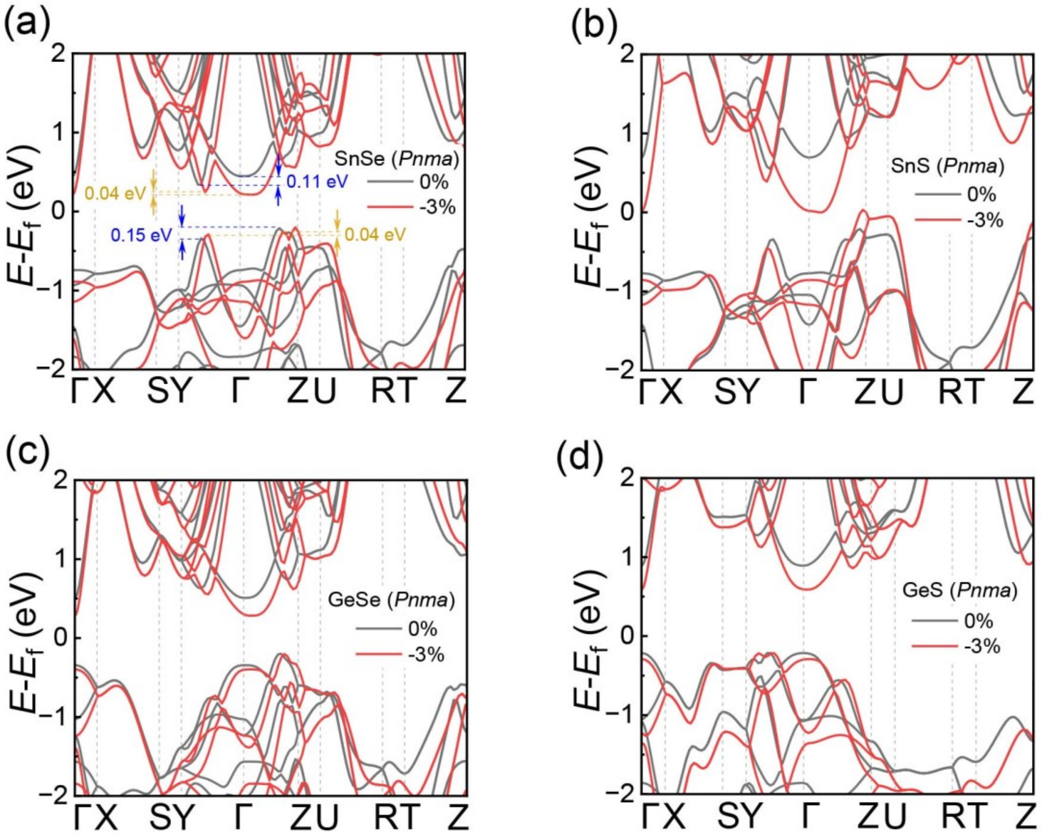

Figure S7. Comparisons of band structures with 0% and -3% strain. (a) SnSe (space group: *Pnma*), (b) SnS (space group: *Pnma*), (c) GeSe (space group: *Pnma*), and (d) GeS (space group: *Pnma*).

4. Determination of carrier relation times

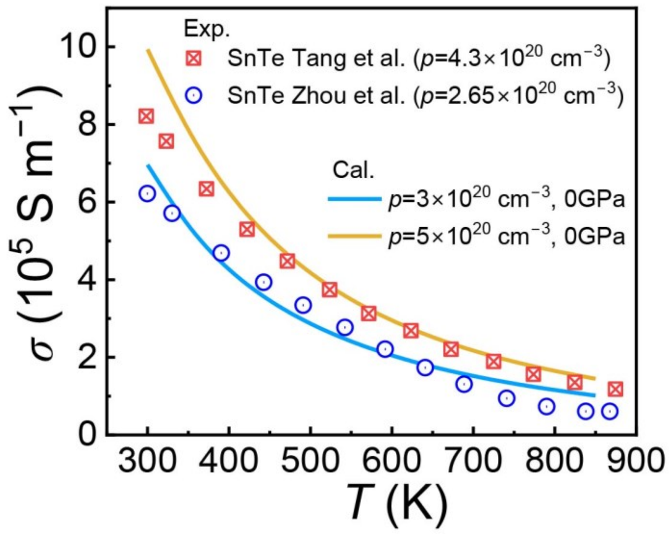

Figure S8. Temperature-dependent electrical conductivity, σ. The curves are theoretically calculated results based on the deformation potential method; dots are the experimental results from the literatures.[15c, 17]

5. Thermoelectric properties

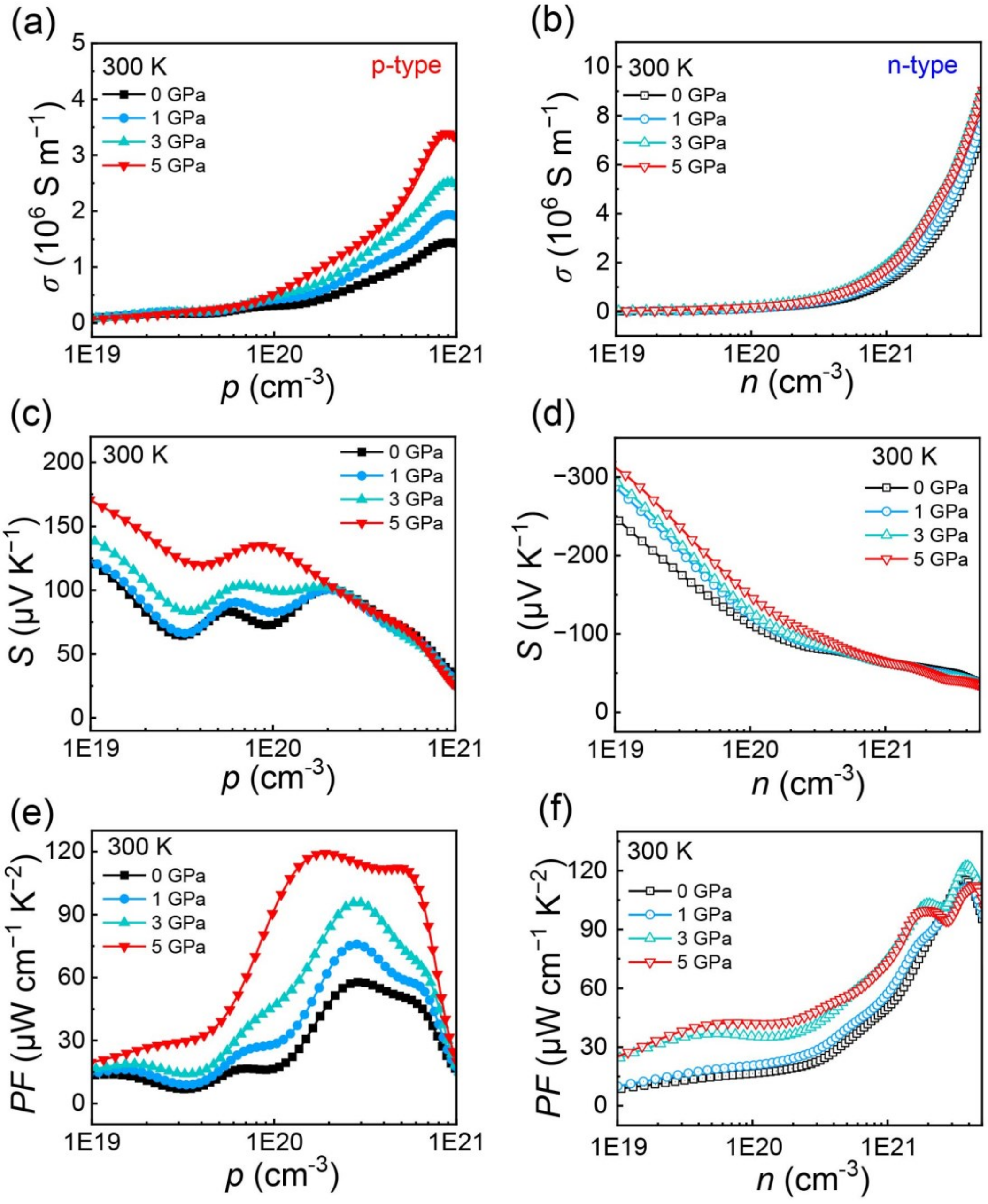

Figure S9. Calculated thermoelectric properties of SnTe under different pressures as a function of carrier concentration (*p* for holes and *n* for electrons) at 300 K. (a-b) electrical conductivity, σ; (c-d) Seebeck coefficient, *S*; (e-f) power factor, *PF*.

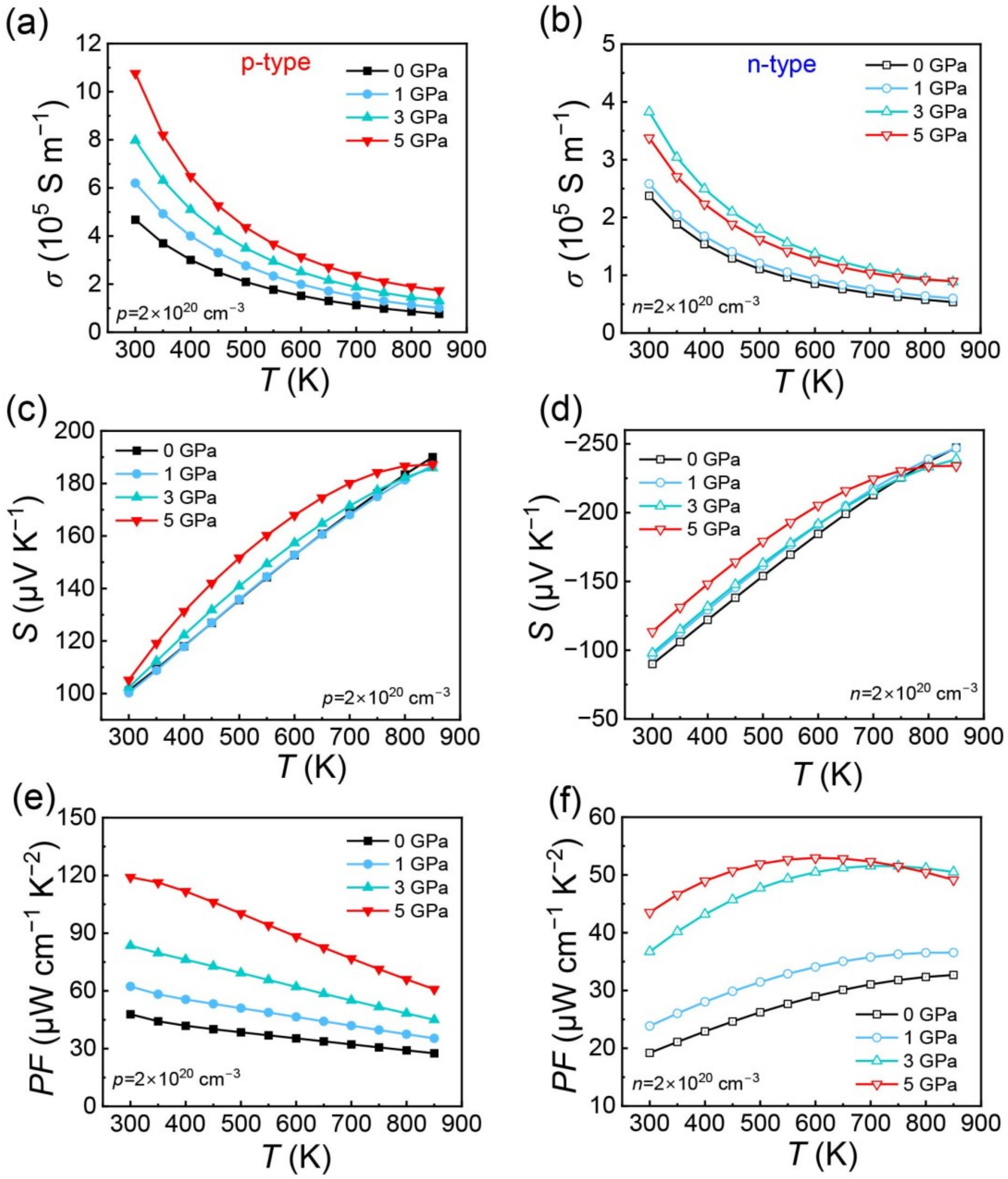

Figure S10. Calculated thermoelectric properties of SnTe under different pressures as a function of temperature, with a fixed carrier concentration $p = 2 \times 10^{20}$ cm^{-3} and $n = 2 \times 10^{20}$ cm^{-3}. (a-b) electrical conductivity, σ; (c-d) Seebeck coefficient, S; (e-f) power factor, PF.

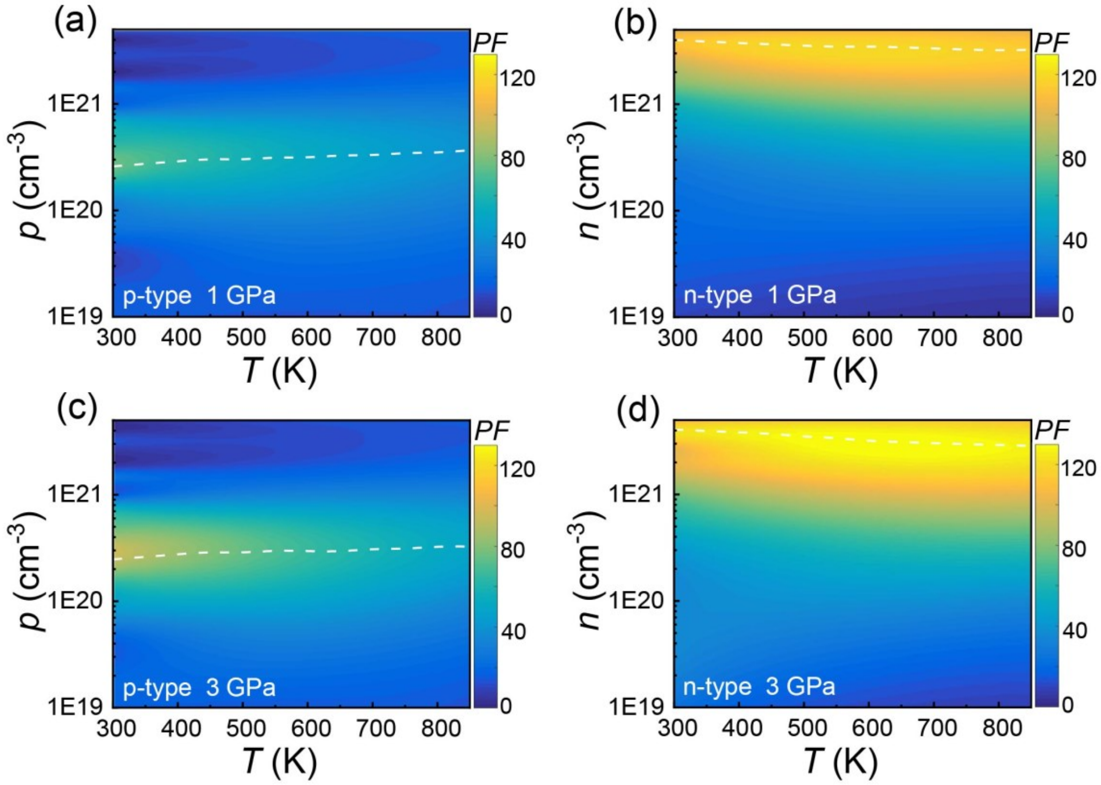

Figure S11. Variations of the power factor (*PF*) with carrier concentration and temperature of SnTe; the different colors represent the magnitude of *PF* in units of μW cm^{-1} K^{-2}. (a) p-type and (b) n-type of SnTe under 1 GPa; (c) p-type and (d) n-type of SnTe under 3 GPa. The white dashed lines represent the optimal carrier concentration (p_{opt} for holes and n_{opt} for electrons) necessary to achieve the PF_{max}.

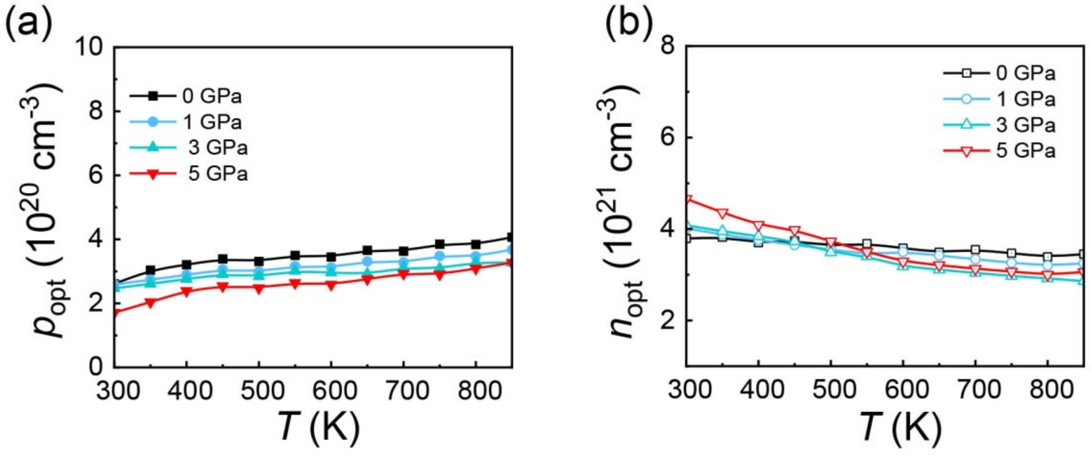

Figure S12. Temperature-dependent optimum carrier concentration (p_{opt} for holes and n_{opt} for electrons) is required to achieve PF_{max}. (a) p_{opt} and (b) n_{opt} for SnTe under 0, 1, 3, and 5 GPa.

6. Phonon transport characteristics

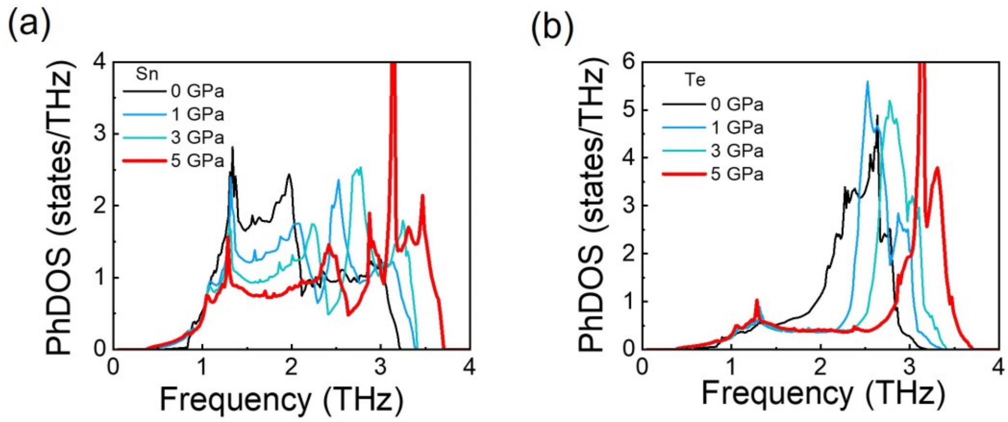

Figure S13. Phonon density of states (PhDOS) of (a) Sn and (b) Te in SnTe under different pressures.

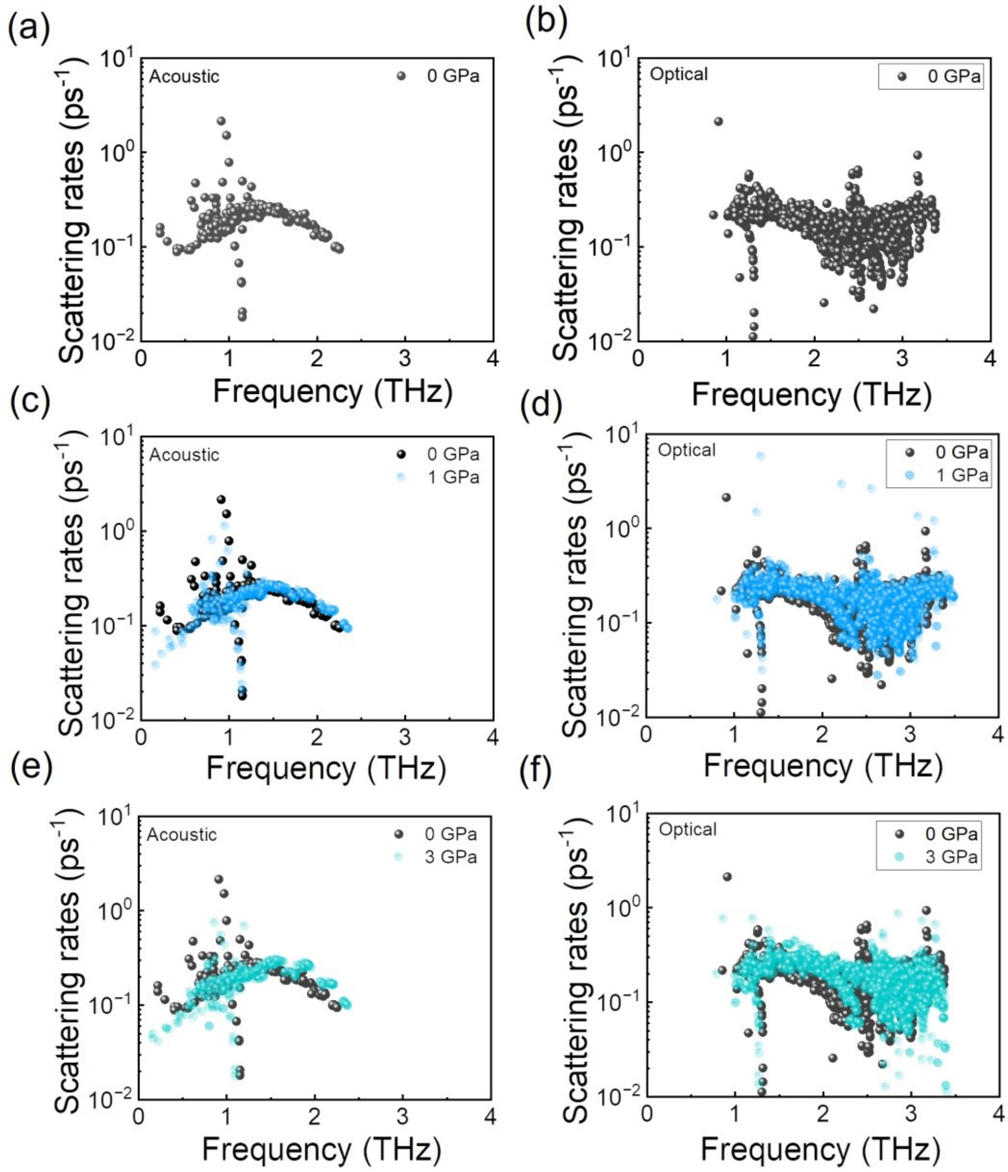

Figure S14. Calculated (a) acoustic and (b) optical phonon modes scattering rates for SnTe under 0 GPa. Calculated (c) acoustic and (d) optical phonon modes scattering rates for SnTe under 0 and 1 GPa. Calculated (e) acoustic and (f) optical phonon modes scattering rates for SnTe under 0 and 3 GPa.

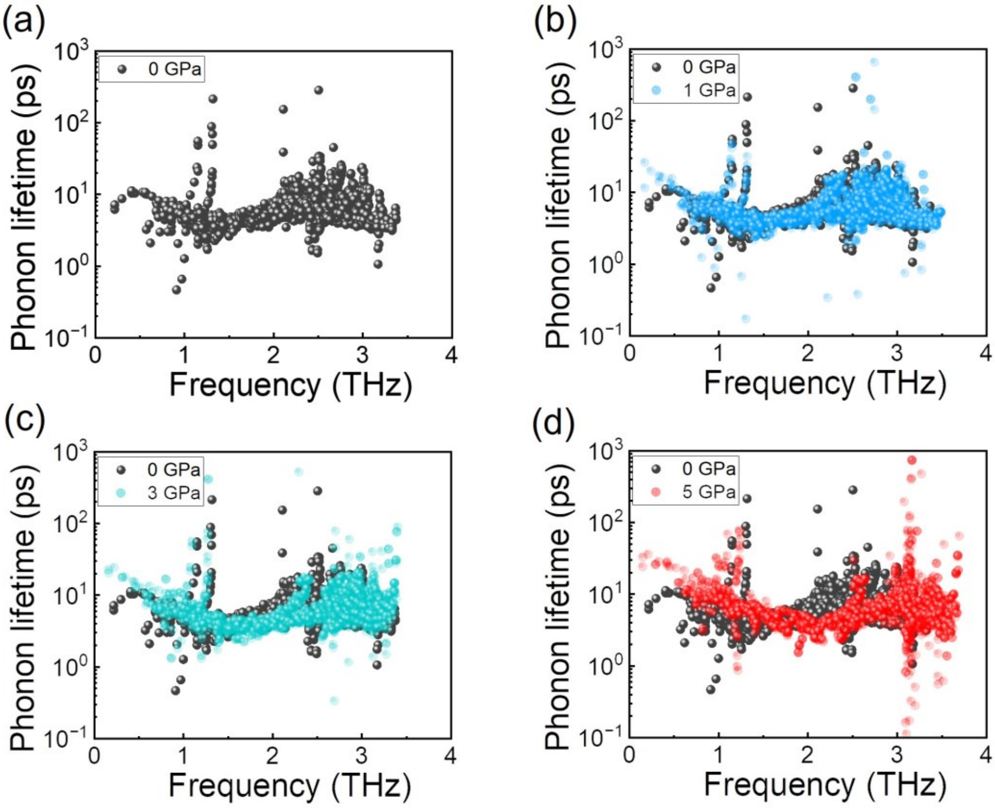

Figure S15. Calculated phonon lifetime of SnTe under (a) 0 GPa, (b) 1 GPa, (c) 3 GPa, and (d) 5 GPa. The phonon lifetime of SnTe under 0 GPa was also provided in Figures S11b-d for comparison.

7. Temperature-dependent lattice thermal conductivity

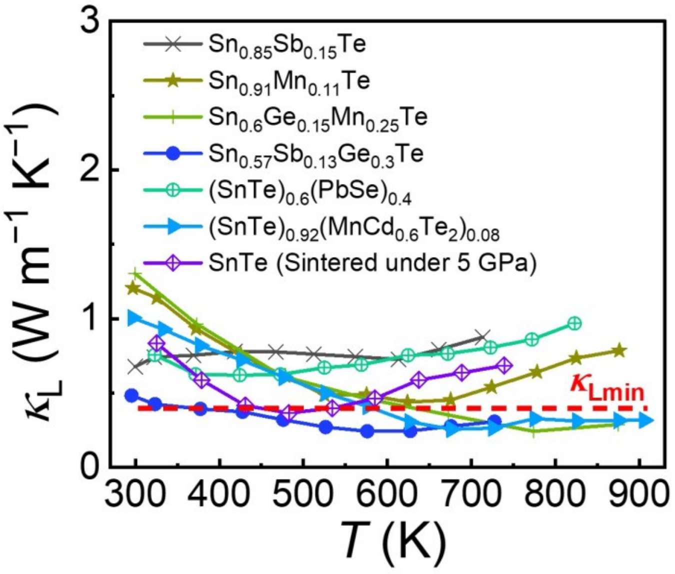

Figure S16. Comparison of the experimental temperature-dependent lattice thermal conductivity from the literature.[15d, 17-18]

8. Figure of merit

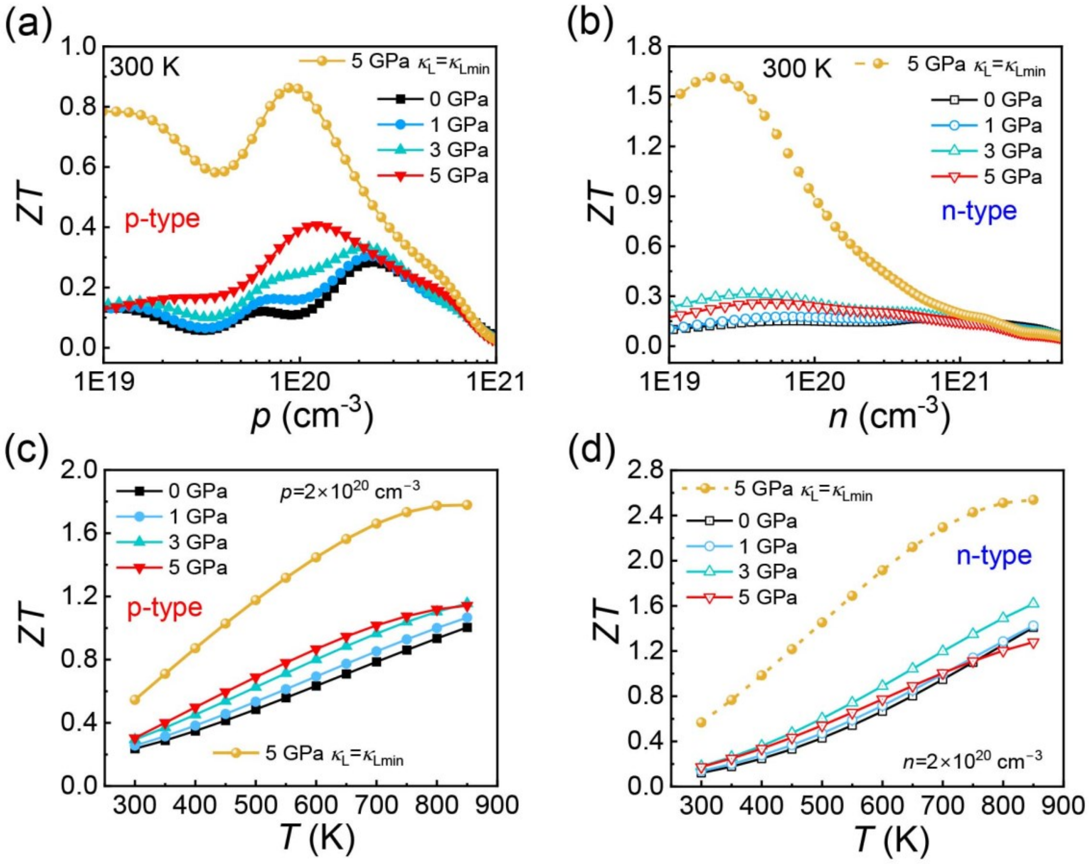

Figure S17. *ZT* of SnTe under different pressures. (a) Variation of *ZT* with hole carrier concentration (p) at 300 K. (b) Variation of *ZT* with electron carrier concentration (n) at 300 K. (c) Temperature-dependent *ZT* of p-type SnTe with a fixed $p = 2 \times 10\ \text{cm}^{-3}$. (d) Temperature-dependent *ZT* of n-type SnTe with a fixed $n = 2 \times 10\ \text{cm}^{-3}$.

References

[1] P. E. Blochl, *Phys Rev B Condens. Matter.* **1994**, *50*, 17953-17979.

[2] K. B. John P. Perdew, Matthias Ernzerhof, *Phys. Rev. Lett.* **1996**, *77*, 3865.

[3] V. Wang, N. Xu, J.-C. Liu, G. Tang, W.-T. Geng, *Comput. Phys. Commun.* **2021**, *267*, 108033.

[4] P. V. C. Medeiros, S. Stafström, J. Björk, *Phys. Rev. B* **2014**, *89*, 041407 (R).

[5] S. Maintz, V. L. Deringer, A. L. Tchougréeff, R. Dronskowski, *J. Compu. Chem.* **2016**, *37*, 1030-1035.

[6] M. Kawamura, *Compu. Phys. Commun.* **2019**, *239*, 197-203.

[7] G. K. H. Madsen, D. J. Singh, *Compu. Phys. Commun.* **2006**, *175*, 67-71.

[8] a Z. Ti, S. Guo, X. Zhang, J. Li, Y. Zhang, *J. Mater. Chem. A* **2022**, *10*, 5593-5604; b T. Jia, J. Carrete, Z. Feng, S. Guo, Y. Zhang, G. K. H. Madsen, *Phys. Rev. B* **2020**, *102*, 125204; c Z. Rashid, A. S. Nissimagoudar, W. Li, *Phys. Chem. Chem. Phys.* **2019**, *21*, 5679-5688.

[9] A. Togo, I. Tanaka, *Scripta Mater.* **2015**, *108*, 1-5.

[10] A. Carreras, A. Togo, I. Tanaka, *Compu. Phys. Commun.* **2017**, *221*, 221-234.

[11] W. Li, L. Lindsay, D. A. Broido, D. A. Stewart, N. Mingo, *Phys. Rev. B* **2012**, *86*, 174307.

[12] W. Li, J. Carrete, N. A. Katcho, N. Mingo, *Compu. Phys. Commun.* **2014**, *185*, 1747-1758.

[13] M. Radjai, A. Bouhemadou, D. Maouche, *Condens. Matter Phys.* **2021**, *24*, 43702.

[14] T. Katsura, Y. Tange, *Minerals* **2019**, *9*, 745.

[15] a M. Zhou, Z. M. Gibbs, H. Wang, Y. Han, C. Xin, L. Li, G. J. Snyder, *Phys. Chem. Chem. Phys.* **2014**, *16*, 20741-20748; b F. Zhang, S. He, R. Li, L. Lin, D. Ren, B. Liu, R. Ang, *Appl. Phys. Lett.* **2021**, *119*, 172101; c X. Yan, S. Zheng, Z. Zhou, H. Wu, B. Zhang, Y. Huang, X. Lu, G. Han, G. Wang, X. Zhou, *Nano Energy* **2021**, *84*, 105879; d J. Q. Li, S. Huang, Z. P. Chen, Y. Li, S. H. Song, F. S. Liu, W. Q. Ao, *Phys. Chem. Chem. Phys.* **2017**, *19*, 28749-28755.

[16] M. Hong, Z.-G. Chen, Y. Pei, L. Yang, J. Zou, *Phys. Rev. B* **2016**, *94*, 161201(R)

[17] F. Li, X. Liu, S. R. Li, X. F. Zhang, N. Ma, X. J. Li, X. Y. Lin, L. Chen, H. J. Wu, L. M. Wu, *Energy Environ. Sci.* **2023**, *14*, 158-172.

[18] a B. Zheng , T. Chen , H. Sun , M. Yang , B. Yang , X. Chen , Y. Zhang , X. Liu *Chinese Phys. Lett.* **2024**, *41*, 057301; b Z. Liu, Z. Guo, L. Deng, *Inorg. Chem.* **2024**, *63*, 5389-5399; c T. Hong, B. Qin, Y. Qin, S. Bai, Z. Wang, Q. Cao, Z.-H. Ge, X. Zhang, X. Gao, L.-D. Zhao, *J. Am. Chem. Soc.* **2024**, *146*, 8727-8736; d A. Banik, T. Ghosh, R. Arora, M. Dutta, J. Pandey, S. Acharya, A. Soni, U. V. Waghmare, K. Biswas, *Energy Environ. Sci.* **2019**, *12*, 589-595; e A. Banik, B. Vishal, S. Perumal, R. Datta, K. Biswas, *Energy Environ. Sci.* **2016**, *9*, 2011-2019.
